# Patient-safety incidents during COVID-19 health crisis in France: An exploratory sequential multi-method study in primary care

**DOI:** 10.1080/13814788.2021.1945029

**Published:** 2021-07-02

**Authors:** Jean-Pascal Fournier, Jean-Baptiste Amélineau, Sandrine Hild, Jérôme Nguyen-Soenen, Anaïs Daviot, Benoit Simonneau, Paul Bowie, Liam Donaldson, Andrew Carson-Stevens

**Affiliations:** a Department of General Practice, Faculty of Medicine, University of Nantes, Nantes, France; b Medical Directorate, NHS Education for Scotland, Glasgow, UK; c Institute of Health and Wellbeing, University of Glasgow, Glasgow, UK; d School of Health and Social Care, Staffordshire University, Stafford, UK; e London School of Hygiene and Tropical Medicine, London, UK; f Division of Population Medicine, Cardiff University, Cardiff, UK

**Keywords:** Patient safety incident, primary care, COVID-19, lockdown

## Abstract

**Background:**

The COVID-19 pandemic has resulted in the rapid reorganisation of health and social care services. Patients are already at significant risk of healthcare-associated harm and the wholesale disruption to service delivery during the pandemic stood to heighten those risks.

**Objectives:**

We explored the type and nature of patient safety incidents in French primary care settings during the COVID-19 first wave to make tentative recommendations for improvement.

**Methods:**

A national patient safety incident reporting survey was distributed to General Practitioners (GPs) in France on 28 April 2020. Reports were coded using a classification system aligned to the WHO International Classification for Patient Safety (incident types, contributing factors, incident outcomes and severity of harm). Analysis involved data coding, processing, iterative generation of data summaries using descriptive statistical analysis. Clinicaltrials.gov: NCT04346121.

**Results:**

Of 132 incidents, 58 (44%) related to delayed diagnosis, assessments and referrals. Cancellations of appointments, hospitalisations or procedures was reported in 22 (17%) of these incidents. Home confinement-related incidents accounted for 13 (10%) reports and inappropriate medication stopping for five (4%). Patients delayed attending or did not consult their general practitioner or other healthcare providers due to their fear of contracting COVID-19 infection at an in-person visit in 26 (10%) incidents or fear of burdening their GPs in eight (3%) incidents.

**Conclusion:**

Constraints from the first wave of the COVID-19 pandemic have contributed to patient safety incidents during non-COVID-19 care. Lessons from these incidents pinpoint where primary care services in France can focus resources to design safer systems for patients.

KEY MESSAGES

- A national primary care emergency response plan that supports primary care practitioners to organise communication with patients, screen vulnerable patients, and coordinate care in case of cancellations, could have mitigated most of the reported non-COVID-19-related incidents during the first wave.

## Introduction

Healthcare-associated harm is an established threat to public health and a source of avoidable harm to patients. More than 40 million patient safety incidents result in healthcare-associated harm worldwide annually [1]. Half of those patient harms are preventable [2], and almost all avoidable incidents are systemic and are inextricably linked to complex interactions between, for example, people, tasks, technology and organisational factors as well as external influences. The interrelationship between these system-wide factors gives rise to safe or unsafe conditions and outcomes, depending on the context faced at the time.

The COVID-19 pandemic context has resulted in significant disruptions to the delivery of healthcare services worldwide [3,4]. Lockdown announcements and successive governmental measures have caused wholesale disruptions in the routines of patients, providers, and overall care. Notably, equipment and/or beds have been relocated, and consequently, surgeries and procedures have been cancelled or deferred for non-infected patients [5]. Combining these system-wide constraints risks an increase in patient safety incidents [6].

During the COVID-19 first wave, patient safety incidents have continued to occur in primary care settings. Therefore, we aimed to identify the impact of the COVID-19 pandemic on the safety of care delivered to patients with non-COVID-19-related illnesses during the first wave to classify recommendations for system improvement efforts to manage subsequent waves or similar future crises.

## Methods

### Study design and setting

We conducted an exploratory sequential multi-method study of patient safety incidents reported by general practitioners in France during the COVID-19 first wave.

### Data collection

#### .

##### Patient safety incidents inclusion criteria

A patient safety incident is defined as ‘*an event or circumstance that could have resulted, or did result, in unnecessary harm to a patient’* [7]. The study population was patient safety incidents relating to the context of COVID-19 first wave in primary care, not those involving adverse clinical outcomes or complications or treatment of COVID-19 itself.

##### National patient safety incidents reporting platform

We built an online patient safety incident reporting platform, secured by Nantes University Hospital and based on the Royal College of General Practitioners Patient Safety Incident Reporting and Learning Guidance [8] (Supplementary Figures 1–3). The home page defined a patient safety incident and the objectives of the study. A second page collected the characteristics of the reporter (age, department of practice, contact details), and their agreement to participate. A third page gathered data on the incident, including:

- Patient characteristics: age, sex, social deprivation proxied by their health insurance status (*couverture santé solidaire*), patient history/factors that may have favoured occurrence of the incident (free-text field).
- A free-text description of what happened, perceived reasons why the incident occurred, the outcomes and any mitigative actions taken (free-text field).
- judgement about the likelihood of the incident occurring prior to the pandemic (Likert scale).

##### Selection of general practitioners

An email was sent on 28 April 2020 to the contact list of the *Collège National des Généralistes Enseignants (CNGE)*, which represent approximately 16,000 GPs, and inviting them to report patient safety incidents observed since 17 March 2020 (date of the French lockdown announcement). Only one reminder email was sent on 28 May 2020, to prevent over-solicitation of GPs at a hectic time of care delivery. GPs could report multiple incidents. This study covers incidents reported up to 29 June 2020.

### Coder training

A team of six coders (four university GPs and two postgraduate general practice students) was trained to code patient safety incidents according to the methods developed by the PatIent SAfety (PISA) research group (Cardiff University), described below.

### Data coding

Each free-text report was coded using the multi-axial PISA classification system, which is aligned to the World Health Organisation International Classification for Patient Safety, and has been extensively used to characterise patient safety incidents data in primary care [9–13].

The PISA classification includes four coding frameworks, each designed to capture a different aspect of the patient safety incident:

- incident framework to characterise the events leading up to the outcome (*e.g.* delayed diagnosis of an emergency condition).
- contributing factors framework to identify the circumstances, actions or influences reported to have played a part in the origin or development of an incident (*e.g.* video-consultation limiting clinical assessment) [9].
- incident outcome framework to consider the impact on patients, staff and the organisation (*e.g.* emergency surgery).
- severity of harm framework to characterise the level of harm of the outcome (defined as no harm, low, moderate or severe harm, or death) classified in accordance with WHO definitions level [9].

**Figure 1.**
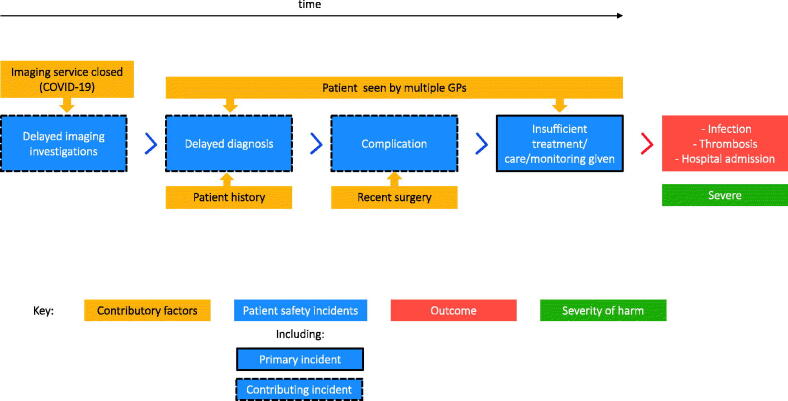
Examples of codes from the classification system using the Recursive Model of Incident Analysis.

Multiple codes for incident types, contributing factors and incident outcomes were applied if necessary (Figure 1). Codes were applied systematically and chronologically according to the nine rules of the Australian Patient Safety Foundation’s Recursive Model of Incident Analysis. Primary incidents included those proximal (chronologically) to the patient outcome, whereas contributory incidents included those that contributed to another incident.

**Figure 2.**
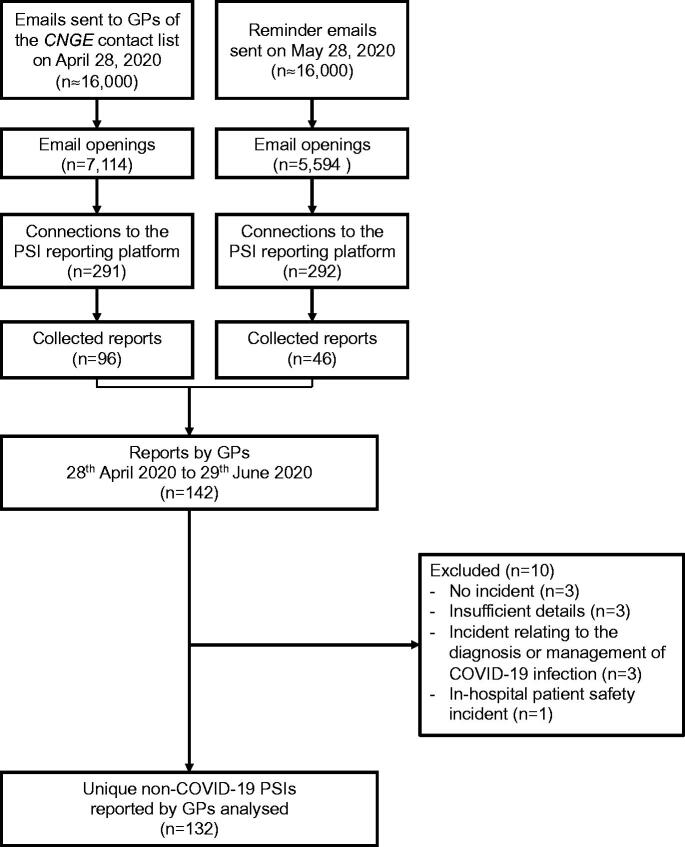
Flow diagram of collected, included and excluded patient safety incident reports. GPs: General Practitioners; CNGE: Collège National des Généralistes Enseignants; PSI: Patient Safety Incident

To clarify details for each submitted case, coders were able to contact reporters to gain additional information. Two independent coders randomly coded each report. In case of disagreement, reports were discussed in team meetings and with an external expert (ACS). Supplemental codes were added in the PISA classification to describe additional insights (type of incidents or contributing factors) when required.

### Analysis

We conducted an exploratory sequential descriptive analysis of coded data. We generated cross-tabulations between incident types, contributory factors and outcomes. Priority areas emerged from the most frequent semantic relationships between incident type and contributory factors. Recommendations to mitigate apparent risks associated with the priority areas were informed by the research team’s awareness of the general practice context in France, iterative searches of the literature, and consultation with topic experts.

### Ethics

The study protocol was prospectively recorded in clinicaltrials.gov (NCT0434612).

Written informed consent was obtained from each participating general practitioner before reporting. This study was approved by the *CNGE* ethics committee, IRB00010804, advice n°27091852200409160. Minor changes were made in incident examples for illustrative purposes and to ensure confidentiality of patients and professionals.

## Results

Of 142 complete reports recorded on the online platform, 132 were included (Figure 2). There were 103 general practitioners reporting between one and four reports (mean: 1.3 ± 0.6). Patients described in the reports were aged between 1 and 95 years old (mean: 56.1 ± 22.8), 74 (57%) were women, and 15 (12%) were considered socially deprived.

The 132 reports described 247 patient safety incidents in total (Table 1). Contributing factors are presented in Table 2. Overall, 285 outcomes (mean: 2.2 per report, Table 3) were identified, with 106 (80%) patient safety incidents having two or more explicit outcomes. Related harms are presented in Table 4.

**Table 1.** Types of 247 patient safety incidents (132 primary incidents and 115 contributing incidents) described in the 132 reports.

Incidents types^a^	Primary incidents^b^ (*n* = 132)	All incidents (*n* = 247)
Diagnosis, assessment & referral	58 (43.9)	96 (38.9)
Diagnosis	45 (34.1)	55 (22.3)
Delayed diagnosis of an emergency condition	18 (13.6)	18 (7.3)
Delay of initial diagnosis of cancer	6 (4.5)	7 (2.8)
Delayed diagnosis (unspecific)	17 (12.9)	18 (7.3)
Other	0 (0.0)	12 (4.9)
Process of assessment	8 (6.1)	27 (10.9)
Delayed assessment	4 (3.0)	9 (3.6)
Errors in the process of identifying patients with acute or serious conditions	3 (2.3)	8 (3.2)
Other	1 (0.8)	10 (4.0)
Incorrect referral	4 (3.0)	10 (4.0)
Other	1 (0.8)	4 (1.6)
Treatment and procedures	43 (32.6)	50 (20.2)
Delayed treatment	22 (16.7)	22 (8.9)
Home confinement complications	13 (9.8)	15 (6.1)
No treatment/care given	6 (4.6)	7 (2.8)
Other	2 (1.6)	6 (2.4)
Investigations	8 (6.1)	28 (11.3)
Diagnostic imaging investigations	3 (2.3)	16 (6.5)
Delayed imaging investigations	1 (0.8)	11 (4.5)
Other	2 (1.6)	5 (2.0)
Laboratory investigations	3 (2.3)	7 (2.8)
Other investigations	2 (1.6)	5 (2.0)
Administrative	3 (2.3)	32 (13.0)
Errors in managing healthcare appointments	1 (0.8)	18 (7.3)
Secondary care appointments	1 (0.8)	15 (6.1)
Other	0 (0.0)	3 (1.2)
Inability to reach out physician	2 (1.6)	7 (2.8)
Incorrect or inefficient transfer of patient information across healthcare systems	0 (0.0)	5 (2.0)
Other	0 (0.0)	2 (0.8)
Medication	16 (12.1)	21 (8.5)
Medication stopped	5 (3.8)	5 (2.0)
Other	11 (8.3)	16 (6.5)
Communication errors	1 (0.8)	15 (6.1)
Between professionals and patients	1 (0.8)	9 (3.6)
Between professionals	0 (0.0)	5 (2.0)
Other	0 (0.0)	1 (0.4)
Other	3 (2.3)	5 (2.0)

^a^Values presented as *n* (%). Primary incidents: incidents chronologically closest to the outcome of the incident for the patient. Contributing incidents: incidents that contributed to the occurrence of another incident.

**Table 2.** Contributing factors underpinning the 132 primary patient safety incidents.

Contributing factors types^a^	Total (*n* = 263)
Patient factors	163 (62.0)
Pathophysiological factors	91 (34.6)
Previous health/medication history	24 (9.1)
Patient confined at home	18 (6.8)
Physical or mental disability	14 (5.3)
Multimorbidity	8 (3.0)
Child	3 (1.1)
Other	24 (9.1)
Patient and/or relatives’ knowledge	36 (13.7)
Fear of contracting Covid-19 in healthcare facilities	26 (9.9)
Fear of burdening General Practitioner	8 (3.0)
Other	5 (1.9)
Behaviour	13 (4.9)
Age	3 (1.1)
Other	20 (7.6)
Organisational factors	86 (32.7)
Reported or cancelled care due to unavailable or closed services	52 (19.8)
Secondary care consultations	19 (7.2)
Imaging	14 (5.3)
Surgery or procedures cancelled/reported	13 (4.9)
Physiotherapist	8 (3.0)
Laboratory	2 (0.8)
Other	5 (1.9)
Continuity of care	14 (5.3)
Between Secondary and Primary Care	9 (3.4)
Others	5 (1.9)
Working conditions	6 (2.3)
Video-consultations	5 (1.9)
Others	8 (3.0)
Healthcare professional factors	10 (3.8)
Equipment factors	1 (0.4)

^a^Values presented as *n* (%).

**Table 3.** Types of outcomes of the 132 primary patient safety incidents.

Outcomes types^a^	Total (*n* = 285)^b^
Unclear outcome	3 (1.1)
No outcome	14 (4.9)
Healthcare professional identified incident and mitigated outcome	5 (1.7)
Patient, relative or carer identified incident and mitigated outcome	2 (0.7)
Other	7 (2.4)
Patient clinical outcomes	122 (42.8)
Pathophysiological or disease-related outcomes	79 (27.7)
Discomfort/pain	11 (3.9)
General deterioration/progression of condition	25 (8.8)
Other	41 (14.4)
Psychological distress	25 (8.8)
Anxiety	6 (2.1)
Psychological difficulty requiring treatment	6 (2.1)
Other	12 (4.2)
Death	8 (2.8)
Other	10 (3.5)
Patient non-clinical outcomes	124 (43.5)
Delays in management (assessment or treatment)	69 (24.2)
Hospital admission	36 (12.6)
Emergency department or casualty	8 (2.8)
Emergency surgery	8 (2.8)
Hospital admission (unspecific)	20 (7.0)
Additional monitoring required	6 (2.1)
Other	13 (4.6)
Organisational outcomes	13 (4.6)
Primary care staff outcomes	9 (3.2)

a Values presented as *n* (%).

b Some incidents have generated several outcomes.

**Table 4.** Harm severity of the 132 primary patient safety incidents.

Harm severity^a^	Primary incidents (*n* = 132)
Unclear harm	33 (25.0)
Unharmful incidents	11 (8.3)
No harm	5 (3.8)
No harm due to mitigating action	6 (4.5)
Harmful incidents	88 (66.7)
Low harm	19 (14.4)
Moderate harm	29 (22.0)
Severe harm	30 (22.7)
Deaths	10 (7.6)

^a^ Values presented as *n* (%).

### Delayed diagnoses and assessments (n = 96)

#### .

##### Emergency conditions

Assessments were delayed for emergency conditions such as stroke (*n* = 5, Box 1 (Example 1)), myocardial infarction (*n* = 1), fracture (*n* = 4, Box 1 (Example 2)), and complicated gastrointestinal infections (*n* = 4).

Box 1 Free-Text Examples of Key Incidents

**Example 1**

Visual concern on the evening of mid-April; starting with a feeling of cold and pain in the right frontoparietal area [lasting] 3–4 days, then persistent visual disturbance on the left visual field; some dizziness […]; the patient thought he had hypoglycaemia (with a feeling of dizziness) and did not consult [me] until end of April for his ‘renewal of treatment’. Patient ‘didn't want to burden’ me (thinking I was overwhelmed in the context of COVID-19). Cerebral scan [… then] MRI: subacute stroke, […] therefore probably irreversible sequelae (homonymous hemianopia). Contributing factor: patient history, social context.

**Example 2**

The patient fell on her wrist: there was a clinical fracture, and the patient was completely unable to use her arm. Rural area. Usually, we have access to an imaging practice 10 km from the medical home and if [fracture] not displaced, we plaster (resin) or [use] thermoformed orthosis in the medical home. Because of the confinement, the local imaging practice was closed and orientated us to another practice 30 km away. So, I sent the patient to the emergency service for imaging and treatment since the delays would be too long to carry out the usual outpatient care. I called the emergency service to let them know. But I learned the same evening when I called the patient back that she did not go there because she was afraid of encountering COVID. I called her daughter and her granddaughter to ensure she’d be taken care of the next day. Delayed treatment with no consequences since there was no vascular, neuro or cutaneous harm from the fracture in the end.

**Example 3**

Patient with history of carcinoma, CLL, type 2 diabetes. In December 2019, I requested an ultrasound scan and a surgical opinion for new lesions and lymphadenopathies following surgery. Ultrasound in January 2020 confirmed the suspect nature of the lymphadenopathies. Appointments with the surgeon (end of January) and the oncologist (early February 2020) were scheduled. CT-scan, biopsy and hospital appointments also scheduled. Letters received. Then nothing… Lockdown began. Mid-April 2020, I saw the patient again for transient diplopia. I tried to get the CT-scan report, but the practice was unreachable and I learned that there has been a PET-scan of which I didn't have the results since the service was also unavailable. I managed to reach the oncology [department], which did not have the results either and realised that the patient had not been summoned for [her] hospitalisation. She also had great difficulty in processing the results, which showed a melanoma with numerous distant metastases. HUGE communication problems related to two causes: general practitioners are not systematically recipients of complementary exams and the confinement has complicated the follow-up of patients.

**Example 4**

Cerebral MRI scan prescribed by an ophthalmologist for decreased visual acuity revealing compressive meningioma. Following appointment with the ophthalmologist was scheduled but his secretary cancelled this without proposal of another appointment or recall. Lack of vigilance from specialist who prescribed the MRI and/or wrong instructions were given to his secretary [resulting in an] outright cancellation of an appointment. The patient came to my consultation not knowing what to do. I quickly referred her to a neurosurgeon considering surgery but with a much longer delay, given the context. Surgery was postponed to the end of June due to the health context (according to the neurosurgeon's report). She told me that she had completely lost vision in her right eye and more than half of her left visual field. She [now] needs permanent assistance of a third person.

**Example 5**

Patient who had an elbow dislocation before confinement. He kept his splint on for four weeks which was too long. As a result, he had his elbow locked at 90 degrees. He called several physiotherapists who could not see him *via* a consultation because physiotherapy practices were closed. He was left to himself without a follow-up consultation and no access to specialist advice. He did not call the general practitioner for advice because he did not want to disturb the general practitioner during the pandemic. When he consulted three weeks later, he was still fixed at 90 degrees. Despite the confinement and [thanks to] my call for a physiotherapist, he started rehabilitation which will take a very long time. This poses considerable risks for a young patient and longer sick leave from work is expected now.

**Example 6**

A patient with anxiety, depression, alcohol dependence, and history of physical violence was socially isolated from family during confinement. As a result, the patient developed increased anxiety, increased alcohol consumption, and became irritable and admitted verbal abuse of the primary caregiver at home. [The patient was not *a priori *identified by the general practice as being vulnerable and at risk of health deterioration due to the confinement.] Anxiolytic treatment, psychotherapy and follow-up by close video consultations [were arranged after delays].

Patients (and their relatives) delayed attending or did not consult their general practitioner and/or emergency departments for fear of contracting COVID-19 (*n* = 9, Box 1 (Example 1)), and also expressed the fear of burdening their general practitioner, thinking that healthcare services were overwhelmed by COVID-19-related care (*n* = 7, Box 1 (Example 2)).

Video consultations complicated the assessment in two reports (general practitioner failed to diagnose atrial fibrillation leading to a stroke; another could not examine a fractured wrist). Physicians overlooked common infections and focussed on COVID-19 as their top differential diagnosis for two patients with serious infections and fever.

### Cancer

Ten other incidents concerned cancer diagnosis (including seven initial diagnoses and two diagnoses of cancer progression). Healthcare services were unavailable, or consultations were delayed (*n* = 8, Box 1 (Example 3)). Three patients with suspected breast cancer had their mammograms and biopsies postponed because the imaging practices were closed. Likewise, CT-scans for two gastrointestinal cancers had been postponed. Surgery was postponed for a patient with a suspicious skin lesion and another patient deteriorated due to postponement of prostate surgery.

### Other conditions

Eighteen other incidents related to delayed diagnoses. From a patient perspective, the fear of contracting COVID-19 was also apparent (*n* = 7), the fear to burden their general practitioner (*n* = 3), and the misunderstanding of home confinement prohibited contact with medical services (*n* = 3).

Video consultation (*n* = 2) created difficulties for diagnosing deep vein thrombosis or pyelonephritis. Closed health services (*n* = 11, Box 1 (Example 4)) resulted in delayed secondary care consultations (gastroenterologist for anaemia investigation, orthopaedic surgeon for suspected hip dysplasia, closed service of maternal and child protection for pregnancy follow-up, nephrologist for severe hyperkalaemia following severe renal failure, *n* = 4) or timely imaging (ultrasound for pyelonephritis, CT-scan for investigation of lymphadenopathy, *n* = 5).

### Delayed treatment (other than medication, n = 22)

Clinical treatment (including physiotherapy), procedures, and surgeries have been delayed – either postponed or cancelled – pending the end of lockdown (Box 1 (Examples 5 and 6)). Services were not available for 18 patients (82%). Most of these incidents resulted in delays in managing patients (*n* = 9, 41%). One patient died from a myocardial infarction that was managed too late because he had a fever and staff were waiting for COVID-19 test results before treating him. Another patient was known to suffer from epilepsy, was socially isolated and died at home from alcohol withdrawal during lockdown (his rehab hospitalisation being postponed *sine die*).

### Home confinement-related incidents (n = 15)

Home confinement-related incidents involved patients typically with history of anxiety or depression (*n* = 7), history of addiction (*n* = 3), advanced age (*n* = 4), or cognitive impairment (*n* = 2, Box 1 (Example 6)). For seven of these patients, home confinement led to incidents in connection with family issues through two main opposing mechanisms: (i) isolation from family/relatives/carers (*n* = 4, leading to disorientation or failure to cope, aggravation of anxiety disorder and/or depression, addiction relapse), or (ii) strict home confinement leading to aggravation of intrafamilial violence (*n* = 3). Twelve of those incidents were harmful; two (13%) of them led to deaths by suicide and failure to cope, three (20%) required hospitalisations, and six (40%) necessitated an introduction or adaptation of psychotropic medication to prevent further harm.

### Medication-related incidents (n = 21)

Medication-related incidents accounted for 21 (9%) incidents. Reasons for inappropriate stopping of medications (*n* = 5) included a patient’s own decision to stop immunosuppressants or chemotherapy for fear of contracting COVID-19 (*n* = 2), stopping of non-steroidal anti-inflammatory drugs (self-decision of patient or advice of a physician, *n* = 2), an inability to pay for medication because of expired insurance-related rights (*n* = 1), insufficient sharing of information between hospital and primary care professionals after hospital discharge (*n* = 1), or due to a strict interpretation of home confinement where a patient refused to attend a pharmacist or general practitioner (*n* = 4).

## Discussion

### Main findings

Whilst efforts to cope with suspected COVID-19 patients were underway; we identified delays in diagnosis, assessments, referrals and treatments for patients with non-COVID-19 health problems, as well as complications arising from home confinement. Patients’ fear of contracting COVID-19 in primary care facilities, and their fear of burdening their general practitioners, have contributed to their poor care and related health outcomes.

### Strengths and limitations

The COVID-19 pandemic sparked an international collaboration between academic general practitioners in France and international patient safety experts. We have used a structured and validated approach, aligned to the WHO International Classification for Patient Safety, to investigate the impact of the COVID-19 pandemic on patients seeking primary care services for non-COVID-19 illnesses.

There is no established patient safety incident reporting culture in France, therefore, we were highly heartened that 103 general practitioners took the time to describe between one and four patient safety incidents. That said, participation of general practitioners was still lower than expected, which may reflect a phenomenon of excessive solicitation *via* surveys in the context of COVID-19 pandemic. However, these reports have identified learning that would have otherwise been lost in history with no way of honouring those patients that have suffered the consequences of healthcare-associated harm.

Incident reports are notoriously variable in data quality, and to mitigate this we sought to clarify report narratives with general practitioners where there were many uncertainties in the case. Overall, whilst these study results should be considered exploratory, we present essential signals that should be interpreted as opportunities for general practitioners and their teams to improve their processes to mitigate avoidable harm to patients. More detailed investigation of incidents at a local and national level, supported by a systems theory-based framework such as Accimaps [14,15], should explore organisational, regulatory and governmental level contributory factors.

### Interpretation of results in relation to existing literature

Gandhi and Singh cautioned early in the COVID-19 pandemic that delayed ‘diagnosis of acute non-COVID-19 diagnoses’ would be mediated by ‘patients not coming in for evaluation due to infection risk’ [6]. Previous reports from the secondary care setting have illustrated the impact of this patient fear in the increased delay of hospital care for myocardial infarction [16,17], or the paediatric population with emergency conditions [18].

Delayed diagnosis when appointments for imaging or elective procedures were cancelled was particularly harmful to patients with acute conditions and cancer signs or symptoms. We also observed delayed diagnoses as a result of telemedicine use.

Identifying incidents directly related to home confinement questions its strict application among the most vulnerable [19,20].

### Implications for practice and policy

In light of the study results, we propose the following recommendations for system improvement efforts.

#### .

##### Addressing fear of contracting COVID-19 in general practice offices and fear of burdening general practitioners through close communication with patients

Our study results highlight that some patients may have erroneous ideas about infection control management in their general practice offices or the workload of their general practitioners. Improved direct communication to counter and overcome these misconceptions with the population could mitigate this confusion. Related, practices should review and update preferred contact approaches for patients.

##### Addressing prevention of home confinement consequences through proactive and repeated contacts with vulnerable patients

Three weeks after the beginning of lockdown, French general practitioners were advised to proactively get in touch with their ‘most fragile patients with chronic conditions’ [21]. Other guidelines recommended, similarly, to prioritise and proactively contact vulnerable patients (at risk of infection, with uncontrolled chronic disease, or experiencing social needs) to invite them to periodically check in [22]. Our study results suggest that those criteria should be extended to patients with addiction. External assistance to assist general practitioners may be necessary to perform this additional task efficiently [23].

##### Addressing the maintenance and follow-up of necessary care through coordination and communication with secondary care

Our study identified the need for transparent communication between primary and secondary care for referrals and imagine requests. Notably, systems are needed for escalating concerns about patients and the provision GPs to access secondary care opinions *via* teleconferencing.

## Conclusion

Major concerns have been raised about the quality and safety of non-COVID-19 care during the COVID-19 pandemic. The incidents we identified highlight a lack of preparedness for the unprecedented magnitude of the pandemic. Nevertheless, we identified powerful signals from the first wave of the pandemic which pinpoint where primary care services in France can focus resources to design safer systems for patients. In doing so, there is an opportunity to mitigate further similar patient safety incidents during subsequent waves or similar future crises.

## Supplementary Material

Supplemental Material

## Data Availability

JPF can be contacted for access to the dataset underlying the current analysis.
