# Supplementary material for: Patient-safety incidents during COVID-19 health crisis in France: An exploratory sequential multi-method study in primary care
Source: Eur J Gen Pract. 2021 Jul 2;27(1):142–51. doi: 10.1080/13814788.2021.1945029 (PMC8259874; doi:10.1080/13814788.2021.1945029)
Supplement: Supplemental Material [file IGEN_A_1945029_SM8499.docx]

**Supplementary online content**

**Supplementary Figure 1** - Incident report form (page 1)


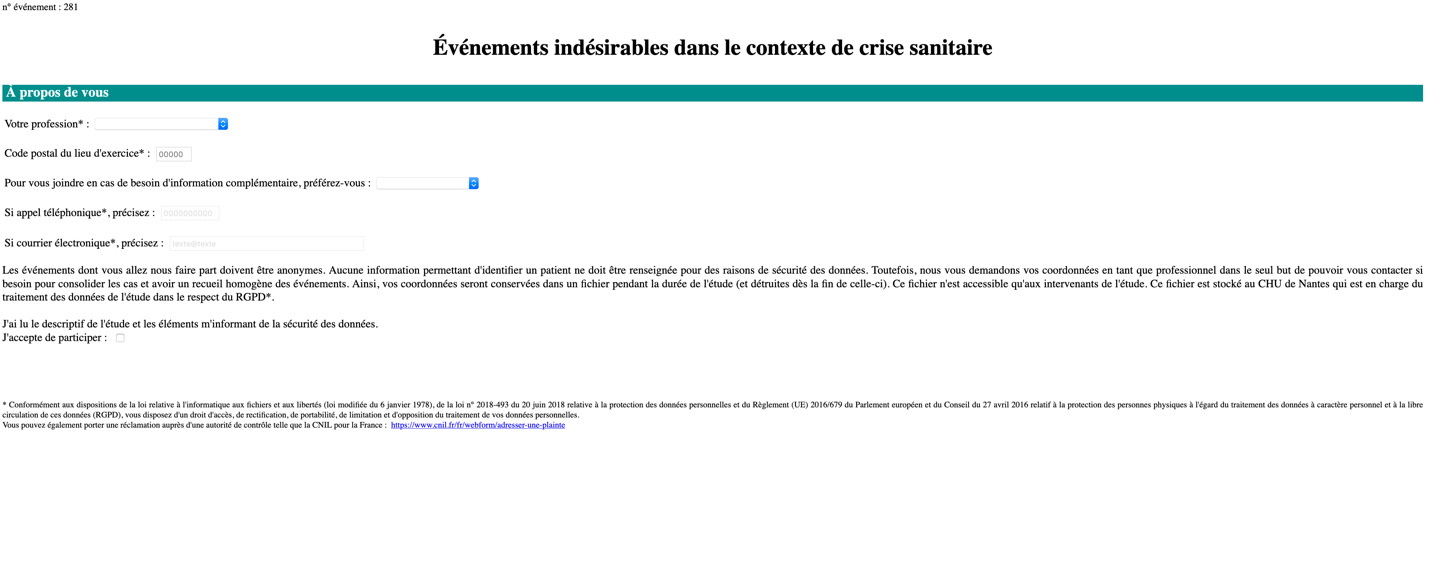


**Supplementary Figure 2** - Incident report form (page 2)
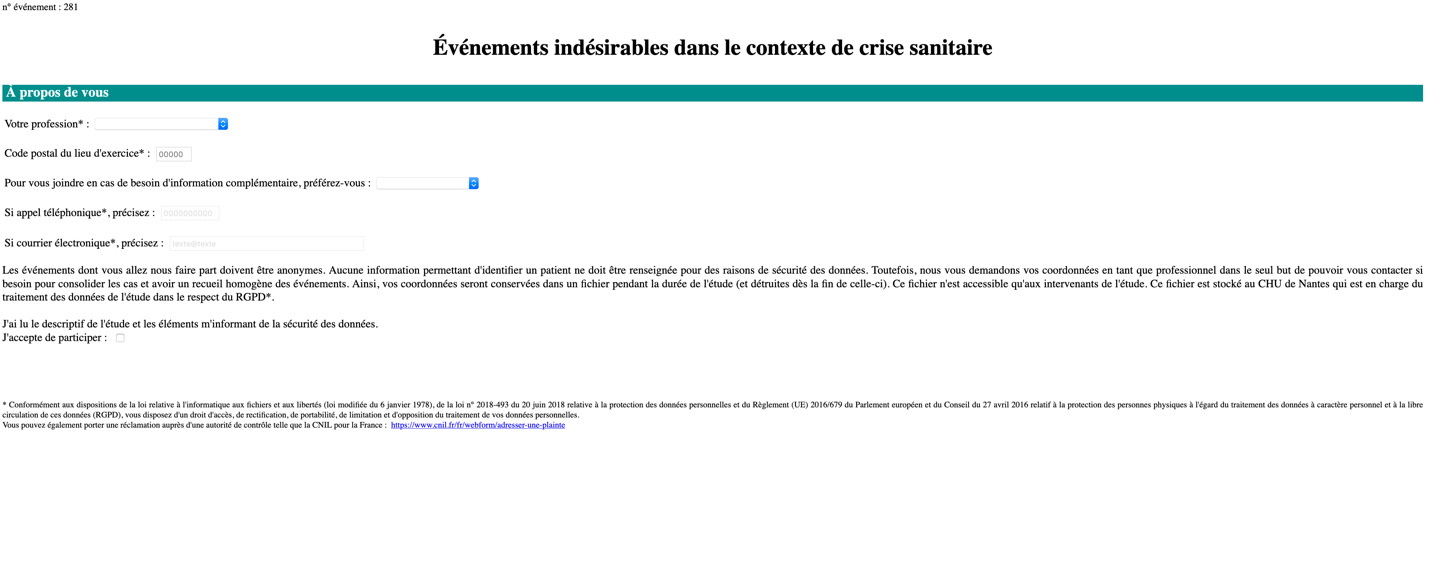


**Supplementary Figure 3** - Incident report form (page 3)


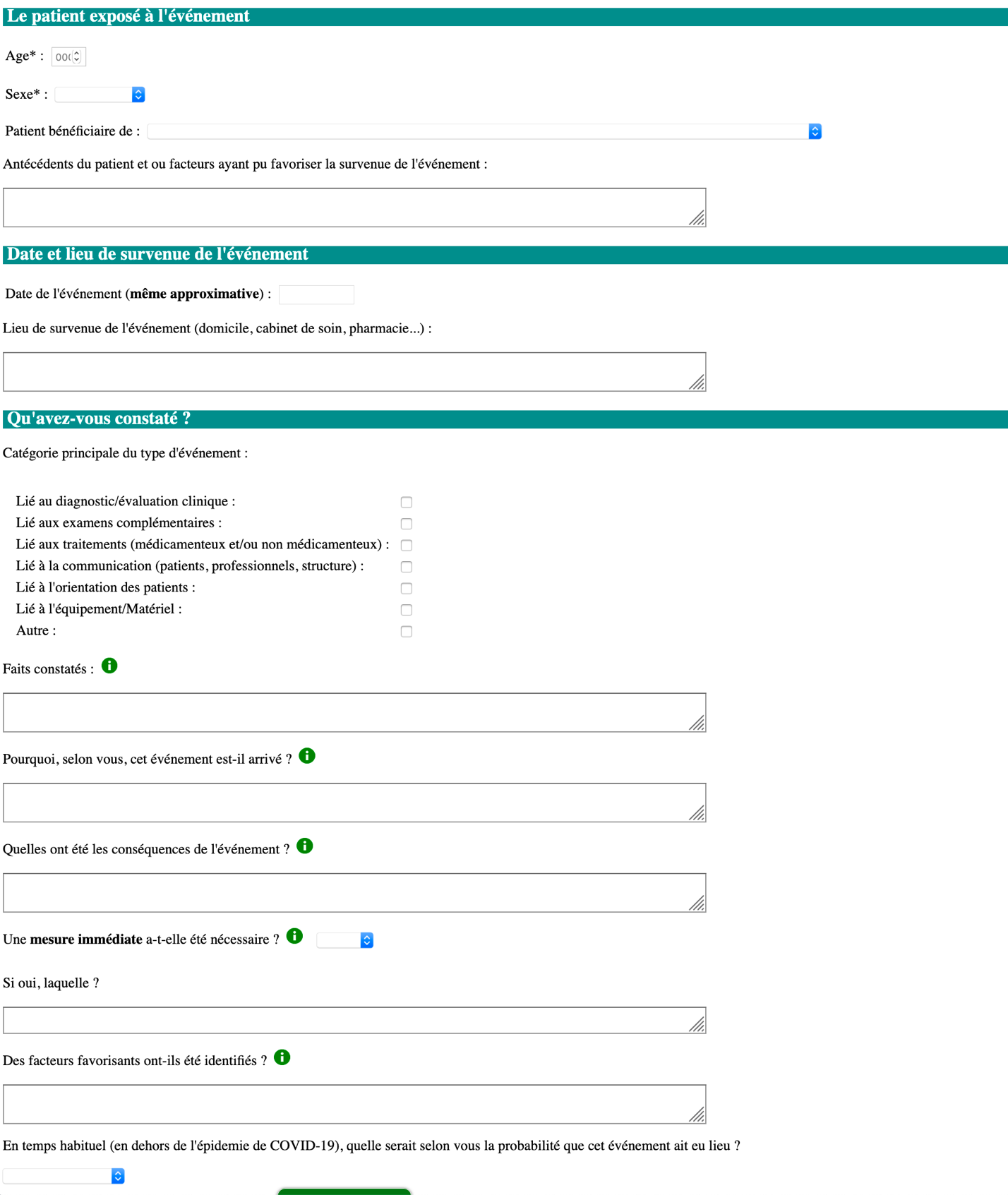


**Supplementary Table 1.** Harm of patient safety incident according to the Primary Care Harm Severity Classification System, 2018^3^

| **Severity** | **Definition** |
| --- | --- |
| No harm | Any incident that ran to completion but no harm occurred to the patient. |
| No harm outcome due to mitigating action | Any incident that had the potential to cause harm to a patient but resulted in no harm. |
| Mild harm | Incident in which: (i) patient was harmed, with mild and short-term impact, on physical, mental or social functioning, that was expected to resolve in a few hours; (ii) patient was harmed but required no or minimal intervention/treatment, e.g. anti-emetic, oral antibiotic or repeat of a minor procedure such as vaccination or insertion of contraceptive implant; and/or (iii) patient or their loved ones experienced transient emotional distress but no long-term consequences and incident report contains words, *e.g.* angry, anxious, confused, distressed, frightened, frustrated, humiliated or upset, that might describe a feeling that occurs at the time of the incident but soon passes. |
| Moderate harm | Incident in which: (i) patient was harmed, causing a medium-term impact on physical, mental or social functioning that was expected to resolve in days; (ii) patient required medical intervention in the form of treatment, e.g. antibiotics or intravenous fluids; (iii) patient required short-term hospitalization for assessment and/or minor treatment in either emergency department or a hospital ward; and/or (iv) patient or their loved ones experienced psychological difficulty of a more longstanding nature but not requiring formal treatment, e.g. as indicated by evidence in the report of more longstanding anxiety, insomnia, or low mood. |
| Severe harm | Incident in which: (i) patient was harmed, causing a major long-term or permanent impact on physical, mental or social function or shortening of life-expectancy; (ii) patient was harmed and required major medical or surgical intervention that, most often, was delivered in a hospital setting, e.g. cardioversion, any major surgery; (iii) patient was harmed and required prolonged hospitalization or admission to coronary care unit, high dependency unit and/or intensive care unit; and/or (iv) patient or their loved ones experienced enduring psychological difficulty that required specialist treatment, e.g. as indicated in the report by evidence of chronic anxiety or depression or psychosis. |
| Death | Incident in which, on the balance of probabilities, death of the patient was caused or brought forward in the short term by the incident. |
| Insufficient detail | Incident for which the report carries insufficient information to evaluate the severity of harm. The report may describe an error or outcome that was not the result of primary health care, e.g. a fall in the waiting room. Alternatively, it may fail to describe any outcome or describe a patient-safety incident but give insufficient information to classify the severity of harm of the outcome, e.g. it may record a delay in getting an appointment but not describe the consequences of the delay for the patient. |

**REFERENCES**

1. Carson-Stevens A, Donaldson LJ. Reporting and learning from patient safety incidents in general practice: a practical guide, 2017. [cited 2021 June 15] Available from: https://www.rcgp.org.uk/-/media/Files/CIRC/Patient-Safety/Reporting-and-learning-from-patient-safety-incidents.ashx?la=en
2. Geneva: World Health Organization. The Conceptual Framework for the International Classification for Patient Safety. Version 1.1. Final Technical Report.; 2009. [cited 2020 November]. Available from: https://www.who.int/patientsafety/implementation/taxonomy/ICPS-report/en/
3. Cooper J, Williams H, Hibbert P, et al. Classification of patient-safety incidents in primary care. Bull World Health Organ. 2018;9:498-505. doi:10.2471/BLT.17.199802
